# Transactions of the First Pan-American Medical Congress

**Published:** 1896-07

**Authors:** 


Transactions of the First Pan-American Medical Congress, held in the City of Washington, D. C., U. S. A., September 5, 6, 7 and 8, 1893. In two volumes. Edited by Charles A. L. Reed, M. D., Secretary-General, Cincinnati, O. Washington: Government Printing Office. 1895.

After a rather protracted delay the transactions of the first Pan-American Congress are now before the profession and present a formidable addition to our stock of medical literature and knowledge. The general scope of the Congress as well as the papers presented at the meetings have been so frequently referred to in these columns that a repetition seems unnecessary. The transactions are carefully edited, the plates and engravings fairly well executed, but the paper and print are considerably below those offered by some of the large eastern publishers. Inasmuch as the Government is the printer and publisher and Congress the distributor, the profession ought to overlook such small defects.

Physicians not belonging to the congress may possibly obtain these transactions by applying to their congressman, and they are well worth a place on the library shelf. W. C. K.

